# Preoperative Management of Pheochromocytoma and Paraganglioma

**DOI:** 10.3389/fendo.2020.586795

**Published:** 2020-09-29

**Authors:** Fang Fang, Li Ding, Qing He, Ming Liu

**Affiliations:** Department of Endocrinology and Metabolism, Tianjin Medical University General Hospital, Tianjin, China

**Keywords:** pheochromocytoma, paraganglioma, preoperative management, catecholamines, adrenergic receptors, α-adrenergic receptor antagonists, hypertension

## Abstract

Pheochromocytoma and paraganglioma (PPGL) are rare neuroendocrine tumors, characterized by excessive release of catecholamines (CAs), and manifested as the classic triad of headaches, palpitations, profuse sweating, and a variety of other signs and symptoms. The diagnosis of PPGL requires both evidence of excessive release of CAs and anatomical localization of CA-secreting tumor. Surgery is the mainstay of treatment for all patients with PPGL unless contraindicated. However, without proper preparation, the release of excessive CAs, especially during surgery, can result in lethal cardiovascular complications. Herein, we briefly reviewed the pathogenesis of this disease, discussed the current approaches and evidence available for preoperative management, summarizing the results of the latest studies which compared the efficacies of preoperative management with or without α adrenergic-receptor antagonists, aiming to facilitate better understanding of the preoperative management of PPGL for the physicians.

## Introduction

A pheochromocytoma is a tumor derived from catecholamine (CA)-producing chromaffin cells in the adrenal medulla, while a paraganglioma is a tumor arising from extra-adrenal chromaffin cells. Since the two tumor types have similar histologic characteristics, they can only be differentiated by anatomical location (intra-adrenal or extra-adrenal). Sympathetic paragangliomas (CAs-producing) derive from paravertebral ganglia of thorax, abdomen, and pelvis, while parasympathetic paragangliomas (rarely produce CAs) arise from vagal and glossopharyngeal nerves at the base of skull and in the neck. Pheochromocytoma and paraganglioma are together referred to as PPGL. Approximately 80%–85% of PPGL are pheochromocytomas, while about 15%–20% are paragangliomas (1). The prevalence of PPGL is about 6 cases per 1 million person-years (2). Almost 5% of patients with adrenal incidentaloma proved to be pheochromocytoma (3). Besides sustained or paroxysmal hypertension and the classic triad of headaches, palpitations, and profuse sweating, a variety of other signs and symptoms may present in PPGL, including tachycardia, fatigue, pallor, nausea, weight loss, and anxiety (4). The biochemical diagnosis of PPGL requires evidence of excessive release of CAs, and then imaging examinations are necessary to detect the anatomical localization of the catecholamine-secreting tumor. The biochemical testing indexes of CAs include CAs (plasma or urine epinephrine-E, norepinephrine-NE, and dopamine-DA), intermediate metabolites of CAs (plasma or urine metanephrine-MN and normetanephrine-NMN), and terminal metabolites of CAs (urine vanillylmandelic acid-VMA). With a mean sensitivity of 97% and a specificity of 93%, the measurements of plasma free MNs are proved by compelling evidence to be the primary test of excessive CAs for diagnosis of PPGL. Therefore, the values of other biochemical tests are limited (1, 5). Once there is clear evidence of excessive CAs, imaging studies should be initiated to locate PPGL. Because of its excellent spatial resolution, computed tomography (CT) should be the first-choice for detection of tumors in thorax, abdomen, and pelvis in most conditions. In patients with metastatic PPGL, an allergy to CT contrast and in whom radiation exposure should be avoided, and for detection of neck and skull paragangliomas, magnetic resonance imaging (MRI) is recommended. ^123^I-metaiodobenzylguanidine (MIBG) scintigraphy, ^18^F-fluorodeoxyglucose (^18^F-FDG) positron emission tomography (PET)/CT scanning, and somatostatin receptor imaging are all functional imaging modalities, and they can be used for occult lesions which failed to be detected by conventional imaging modalities and patients with metastatic PPGL (1). At least one-third of all patients with PPGL carry disease-causing germline mutations, and PPGL may often be part of some hereditary syndromes, such as multiple endocrine neoplasia (MEN) type 2, von Hippel-Lindau (VHL) syndrome and neurofibromatosis type 1 (NF1). Therefore, genetic testing are recommended for all patients with PPGL (6). Surgery is the mainstay of treatment for all patients with PPGL unless contraindicated. And for patients with a hormonally functional PPGL, preoperative management is critical to prevent perioperative complications. The α-adrenergic receptor blockers are the first choice for preoperative management of PPGL (1, 4).

## Catecholamines and Adrenergic Receptors

**Figure 1:**
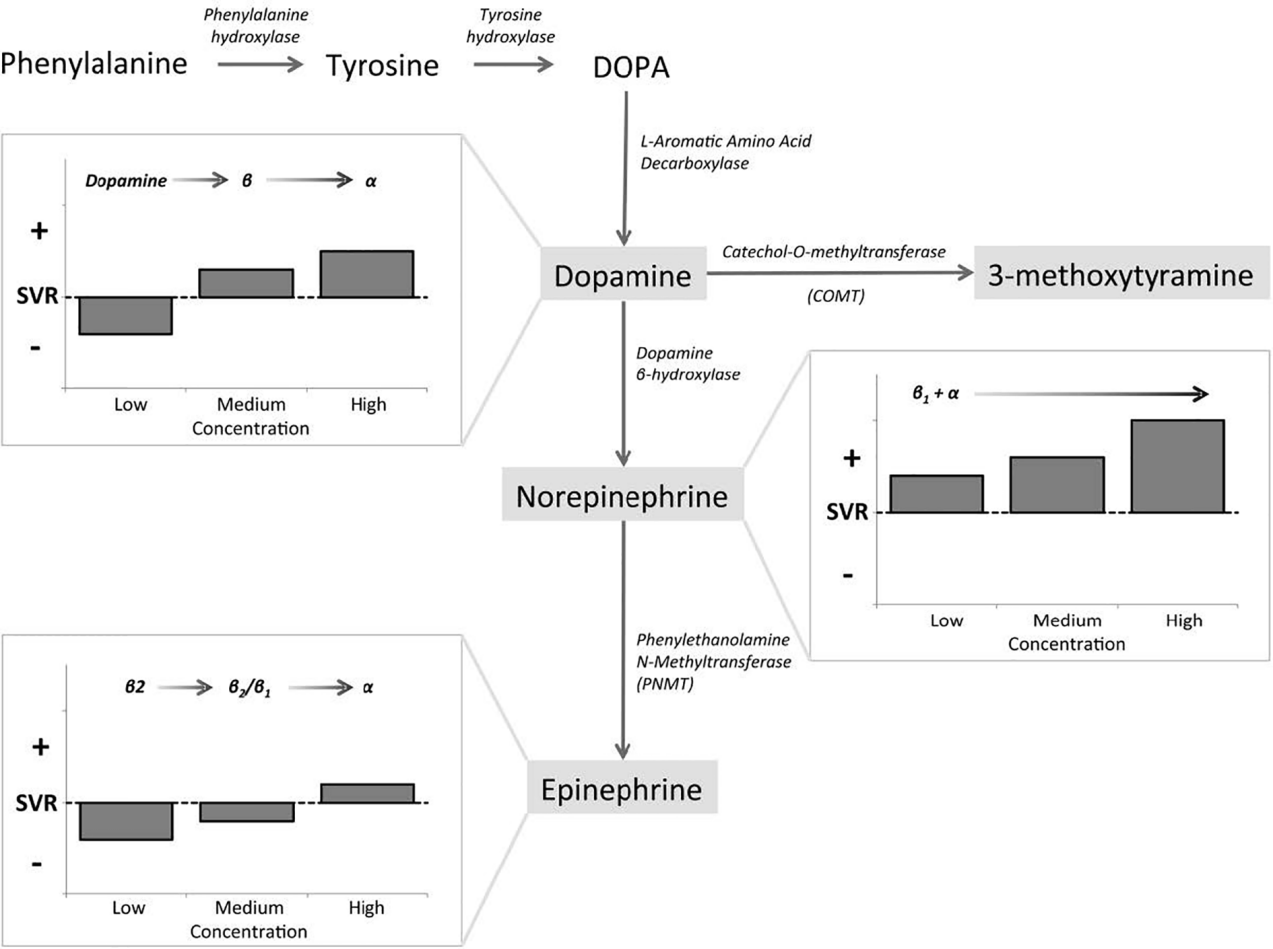
Catecholamines (CAs) synthetic pathway and dose-dependent effects. Epinephrine (E), norepinephrine (NE), and dopamine (DA) exert dose-dependent effects at low, medium, and high circulating concentrations, as governed by affinity to α-AR, β-AR, and DA receptors (10).

The naturally occurring CAs consist of E, NE, and DA, and they are not only important neurotransmitters in the central and peripheral nervous systems, but also play key roles as circulating neurohormones (7). In PPGL, many pheochromocytomas produce both E and NE, and few produces predominantly E, while paragangliomas which arising from extra-adrenal chromaffin cells, produce predominantly NE. In rare cases, sympathetic paragangliomas and pheochromocytomas may produce DA (7, 8). The CAs take effect by interacting with adrenergic receptors (ARs) which express on cell membranes of smooth muscles and visceral organs. Upon binding of CAs to ARs, the signaling pathways are activated, resulting in alterations in smooth muscle tone and organ function (7). There are mainly four kinds of ARs, including α1, α2, β1, and β2. They distribute over different effector organs and tissues, thus mediate different biological responses and correlate with various clinical manifestations (**Table 1**) (8, 9). α1-ARs are primarily expressed at postsynapses of sympathetic nerves and vascular smooth muscle. The activation of α1-ARs causes vasoconstriction, resulting in hypertension. While α2-ARs are primarily localized on presynaptic nerve terminals of sympathetic nerves, resulting in feedback inhibition of NE release. Therefore, activation of α2-ARs dampens the functions of sympathetic nerves. α2-ARs are also expressed at the nonsynaptic sites of vascular smooth muscles, mediating vasoconstriction when activated. β1-ARs are mainly localized in the heart, and they mediate positive inotropic and chronotropic responses, thus resulting in hypertension and tachycardia. β2-ARs are present in smooth muscles of most other organs, such as tracheal and bronchial, gallbladder, and uterus. They mediate relaxation of these smooth muscles, thus playing an important role in spasmolysis. Both α and β1-AR subtypes have high affinity for E and NE, but β2-AR subtype has much higher affinity for E than NE (7). Although these properties of ARs allow us to better understand their functions and response to E and NE, the actual effects/outcomes are much more complex, since the proximity of sites of E and NE release to ARs and the concentrations at effector sites are also significant determinants of AR-mediated actual effects of CAs (8). The effects of CAs on target organs exert a dose-dependent manner by acting on different receptors. NE predominantly acts on α and β1-ARs, and the higher concentrations of NE mediate more severe systemic vascular resistance (SVR), thus patients with PPGL secreting high concentrations of NE may present with sustained hypertension, headache, palpitation, and sweating. For low and medium concentrations of E, β2-ARs, which mediate vasodilatation in skeletal muscle, are the dominant receptors, thus hypotension may manifest. While high concentration of E primarily acts on α-ARs which may cause SVR, patients with predominantly E-secreting PPGL may present with paroxysmal hypertension resulting from different concentrations of E. Moreover, E is an important metabolic hormone, which stimulates lipolysis, thermogenesis, and glycolysis, and plasma glucose concentrations may be increased by stimulating glycogenolysis and gluconeogenesis. Physiological concentration of DA acts primarily on DA receptors, leading to negative cardiac inotropic action and renal artery dilatation. As DA concentration increases, DA can act on α and β-ARs, causing variable degrees of hypertension and tachycardia. Therefore, patients with DA-secreting PPGL may present with different manifestations, varying from hypotension to normotension and hypertension (8, 10, 11). CAs synthetic pathway and effects in dose-dependent manner are shown in **Figure 1** (10). These CA-specific effects on ARs can explain the various presentations of patients with PPGLs and are the foundation for appropriate preoperative management.

**Table 1:** Adrenergic receptors-mediated responses of effector organs (9).

Effector organs	Receptor type	Responses	Most relevant clinical manifestations
Eye			
Radial muscle, iris	α1	Contraction (mydriasis) ++	Blurry vision
Ciliary muscle	β2	Relaxation for far vision +	
Heart			
SA node	β1, β2	Increase in heart rate ++	Palpitations, angina
Atria	β1, β2	Increase in contractility and conduction velocity ++	
AV node	β1, β2	Increase in contractility and conduction velocity +++	
His-Purkinje system	β1, β2	Increase in contractility and conduction velocity +++	
Ventricles	β1, β2	Increase in contractility and conduction velocity, automaticity, and rate of idioventricular pacemakers +++	
Arterioles			
Coronary	α1, α2, β2	Constriction +, dilations ++	Angina
Skin and mucosa	α1, α2	Constriction +++	Pallor
Skeletal muscle	α1, β2	Constriction ++, dilations ++	Hypertension
Cerebral	α1	Constriction (slight)	Stroke
Pulmonary	α1, β2	Constriction +, dilations ++	Edema
Abdominal viscera	α1, β2	Constriction +++, dilations +	E.g., Bowel ischemia
Salivary glands	α1, α2	Constriction +++	
Renal	α1, α2, β1, β2	Constriction +++, dilations +	Renal failure
Veins (systemic)	α1, α2, β2	Constriction ++, dilations ++	Orthostatic hypotension
Lung			
Tracheal and bronchial muscle	β2	Relaxation +	
Bronchial glands	α1, β2	Decreased secretion; increased secretion	
Stomach			
Motility and tone	α1, α2, β2	Decrease (usually) +	Early satiety, discomfort
Sphincters	α1	Contraction (usually) +	
Intestine			
Motility and tone	α1, α2, β1, β2	Decrease +	Constipation, ileus
Sphincters	α1	Contraction (usually) +	
Secretion	α2	Inhibition	Constipation
Gallbladder and ducts	β2	Relaxation +	Gallstones
Kidney			
Renin secretion	α1, β2	Decrease +, increase ++	
Urinary bladder			
Detrusor	β2	Relaxation (usually) +	Urinary retention
Trigone and sphincter	α1	Contraction ++	
Ureter			
Motility and tone	α1	Increase	
Uterus	α1, β2	Pregnant: contraction; relaxation Nonpregnant: relaxation	
Sex organs, male	α1	Ejaculation ++	
Skin			
Pilomotor muscles	α1	Contraction ++	
Sweat glands	α1	Localized secretion +	Sweating
Spleen capsule	α1, β2	Contraction ++, relaxation +	
Skeletal muscle	β2	Increased contractility; glycogenolysis; K^+^ uptake	Hyperglycemia, glycosuria
Pancreas			
Acini	α	Decreased secretion +	
Islet (β cells)	α2	Decreased secretion +++	Hyperglycemia, glycosuria
	β2	Increased secretion +	Hypoglycemia
Fat cells	α2, β1, β2	Lipolysis +++ (thermogenesis)	Feeling warm
Salivary glands	α1	K^+^ and water secretion +	
	β	Amylase secretion +	
Lacrimal glands	α	Secretion +	Lacrimation
Pineal gland	β	Melatonin synthesis	
Posterior pituitary	β1	Antidiuretic hormone secretion	Decreased diuresis

SA, Sinoatrial; AV, atrioventricular. Highest (+++) to lowest (+) intensity of adrenergic nerve activity in the control of various organs and functions. Where (+) is not given, intensity is not specified (8, 9).

## Preoperative Management of PPGL

PPGL is characterized by hypertension and low blood volume, resulting from excessive concentration of CAs in the plasma. With insufficient preoperative antihypertensive management or untreated hypovolemia, the hemodynamic instability during surgical treatment of PPGL may be lethal. Therefore, preoperative management, which includes hypertension control and expansion of blood volume, is extremely important (12, 13). The main goal of preoperative management of PPGL is to normalize blood pressure and heart rate, restore effective circulating blood volume, improve metabolic condition, and prevent a patient from CA storm and hemodynamic instability during surgery (8). However, since the low incidence of PPGL, evidence-based studies (such as large-scale randomized controlled trials-RCTs) comparing different treatments are scarce, and there is still no consensus for the most appropriate preoperative management of PPGL (8, 13). However, the most common approach is to block the function of excessive plasma CAs, and α-AR antagonists are the first choice. β-AR antagonists, calcium channel blockers (CCBs), and CA synthesis inhibitors are also recommended when necessary, but β-AR antagonists can only be used after sufficient pretreatment with α-AR antagonists to avoid unopposed α-AR overstimulation. Brief instructions of preoperative drugs for PPGL are shown in **Table 2**. In the meantime, a high-sodium diet and fluid intake are recommended to reverse CA-induced blood volume contraction to prevent severe hypotension (1, 8).

**Table 2:** Brief instructions of preoperative drugs for pheochromocytoma and paraganglioma (PPGL).

Drugs		Doses	Starting time	Advantages	Adverse effects and points for attention
α-AR antagonists	Phenoxybenzamine	Initially 10mg BID, usually 1mg/kg/d	1–2 weeks before surgery	The effect is profound and long-acting.	Prolonged hypotension postoperatively, orthostatic hypotension, reflex tachycardia, nasal congestion, central sedation
	Prazosin	Initially 0.5–1 mg BID-TID, usually 6–15 mg/d, maximum dose of 20 mg/d		Lower risk of postoperative hypotension, seldom cause reflex tachycardia, nasal congestion and central sedation	Orthostatic hypotension, the anti-hypertensive effect may not as profound as phenoxybenzamine
	Perazosin	Usually 2–10 mg/d, maximum dose of 20 mg/d			
	Doxazosin	Initially 1mg QD, usually 2–8 mg/d, maximum dose of 16 mg/d			
β-AR antagonists	Propranol	Initially 10 mg TID-QID, maximum dose of 200 mg/d	After adequate α-AR blockade		Never be used alone or before adequate α-AR blockade, should not be used for patients with asthma, severe atrioventricular block or bradycardia, sick sinus syndrome, severe heart failure, and cardiogenic shock
	Atenolol	Usually 12.2–25 mg BID-TID			
	Metoprolol	Usually 25–50 mg BID-TID			
	Metoprolol controlled release tables	25–200 mg QD		Long-acting	
CCBs	Nicardipine	Initially 20 mg TID, maximum dose of 120 mg/d	1–2 weeks before surgery if necessary	Do not cause drug-induced orthostatic hypotension and reflex tachycardia, prevention of CA-mediated coronary vasospasm and myocarditis	Monotherapy of CCBs may not be effective enough for patients with biochemically active PPGL, which should be combined with α-AR antagonists.
	Amlodipine	5–10 mg QD			
	Nifedipine	Initially 10mg TID, maximum dose of 120mg/d			
	Nifedipine controlled release tables	30-60mg QD			
CA synthesis inhibitor	Metyrosine	Initially 500 mg/d, maximum dose of 4 g/d	At least 1-3 weeks before surgery	Directly inhibit the CAs biosynthesis	Sedation, somnolence, anxiety, depression, and rarely leading to extrapyramidal signs (such as parkinsonism)

At present, there is no consensus for when preoperative management should be started to ensure adequate preparation for surgery. In most circumstances, α-AR antagonists are usually initiated 1–2 weeks preoperatively to normalize blood pressure, heart rate and to replete contracted blood volume. In patients with CA-induced cardiomyopathy or other organ damage, α-AR antagonists should be start much earlier (8). There is not enough evidence from RCTs to determine the optimal target blood pressure and cardiac condition, and the present recommendations are from institutional experience and retrospective studies. In 1983, Roizen proposed the Roizen criteria to assess for adequate α-AR blockade: 1. No blood pressure >160/90mmHg should be evident for 24 h before surgery; 2. For patients with orthostatic hypotension, readings >80/45mmHg should be present; 3. No ST-T changes is present in electrocardiogram for at least 1 week; 4. No more than one premature ventricular contraction for every 5 min (14). In 2007, the Endocrine Society recommended that the goal is to achieve target blood pressure of less than 130/80 mmHg when sitting and no less than 80/45 mmHg when standing, and target heart rate is about 60–70 beat per minute (bpm) when sitting and 70–80 bpm when standing (8). These targets should be individualized according to age, general conditions and complications. However, it should be clearly noted that complete prevention of perioperative hypertension and tachycardia cannot be achieved by any doses or combinations of drugs (1).

## α-AR Antagonists

Two types of α-AR antagonists are widely used clinically, non-selective and selective α-AR antagonists. Phenoxybenzamine is a non-selective, non-competitive α-AR antagonist, which binds irreversibly with both α1 and α2-AR. Phenoxybenzamine is long-acting and its effects persist long after it has been discontinued, since the effect diminishes only after α-AR resynthesis (15, 16). The starting dose of phenoxybenzamine is usually 10 mg twice a day and the dose can be increased in increments of 10–20 mg every 2–3 days, until the clinical manifestations are well controlled or adverse effects appear. Generally speaking, a total daily dose of 1 mg/kg is adequate for most patients of PPGL, while for some patients, larger doses may be required. Phenoxybenzamine can also be administered 3 days before operation by infusion (0.5 mg/kg/d) for 5 h a day, but this approach requires closely monitoring of the patient (8). The advantage of phenoxybenzamine is that its effect is profound and long-acting, even when excessive amounts of CAs reach the circulation, since the combination of phenoxybenzamine and α-AR is non-competitive and non-selective. Orthostatic hypotension is the common adverse effect of nearly all the α-AR antagonists. Another disadvantage of phenoxybenzamine is the high incidence of reflex tachycardia, as a result of the inhibition of α2-AR which localized in the presynaptic membrane and participate in the negative feedback of NE release. Blockade of the presynaptic α2-AR results in the release of NE into circulation and an increase in chronotropic activity of the heart, leading to tachycardia. Therefore, β-AR antagonist is often required to control tachycardia after the use of phenoxybenzamine. Other side effects related to inhibition of α2-AR include nasal congestion and retrograde ejaculation. Moreover, phenoxybenzamine can cause prolonged hypotension postoperatively due to its long-acting features, which should draw the clinicians’ attention. Since it can pass the blood-brain barrier, it may cause central sedation occasionally (17).

Selective α-AR antagonists, including prazosin, terazosin, and doxazosin, competitively inhibit only α1-AR, and they are short-acting drugs used for patients with PPGL as well. The initial dose of prazosin is 0.5–1 mg, 2–3 times a day, and the dose can be gradually increased to 6–15 mg per day. Terazosin is usually given in doses of 2–10 mg per day. For both prazosin and terazosin, the doses of higher than 20mg per day may not bring extra benefit. Doxazosin, with a half-life much longer than the other selective α-AR antagonists, can be dosed once daily, and is the most commonly used selective α-AR antagonist in recent years. The starting dose of doxazosin is usually 1 mg per day, and the most commonly used doses are 2–8 mg per day. The maximum recommended dose is 16 mg per day (8). Since all the α-AR antagonists may cause orthostatic hypotension, they are recommended to be given just before bedtime when used for the first time, and the patients should be reminded of slow body position changes. Unlike phenoxybenzamine, selective α1-AR antagonists do not cause reflex tachycardia, and seldom cause nasal congestion, because they do not inhibit α2-AR. Due to the nature of competitive inhibition of α1-AR, they have a shorter duration of action and lower risk of postoperative hypotension. Furthermore, they minimally pass the blood-brain barrier, and central sedation is rare. Compared to phenoxybenzamine, selective α1-AR antagonists have fewer side effects. However, as competitive inhibition may be overcome by excessive concentration of CAs, the anti-hypertensive effect is not as profound as phenoxybenzamine (17, 18).

## Non-Selective or Selective α-AR Antagonists?

Evidence from RCTs or systematic reviews comparing the effectiveness of non-selective and selective α-AR antagonists for patients of PPGL is unavailable (1). However, many retrospective and prospective studies have made efforts to compare these two types of drugs. Some studies show that non-selective α-AR antagonists, phenoxybenzamine, and selective α-AR antagonist, doxazosin, have similar effects on controlling blood pressure (16, 19, 20), while some studies report that hypertension is better controlled by phenoxybenzamine than by doxazosin (15, 21, 22). With respect to hemodynamic instability, some studies reveal that phenoxybenzamine is as effective as doxazosin (15, 23). However, most studies report that doxazosin is associated with less adverse effects than phenoxybenzamine, such as reflex tachycardia, postoperative hypotension, edema, and nasal congestion (16, 19, 20, 22). In general, both phenoxybenzamine and doxazosin are able to effectively control perioperative blood pressure and to prevent hemodynamic instability in patients of PPGL. Hypertension may be slightly better controlled by phenoxybenzamine for some patients, at the cost of higher risk of postoperative hypotension. To control reflex tachycardia, phenoxybenzamine more often required co-treatment by β-AR antagonists. While doxazosin is more likely to require additional antihypertensive drugs, such as calcium channer blockers (CCBs) (17), for blood pressure control, and is proved to have much less adverse effects. At present, choice of medication depends on institutional preference whether to use a non-selective or selective α-AR antagonist, because there is still no consensus according to the existing evidences.

## Is α-AR Blockade Necessary for All Patients of PPGL?

Many retrospective studies have reported the use α-AR antagonists as the first-choice to prevent perioperative complications of PPGL, including intraoperative hypertension, tachycardia, and hemodynamic instability (24–28). North American neuroendocrine tumor society consensus guidelines recommended that all patients with PPGL (even those with normal levels of CAs) should use appropriate management to block effects of released CAs (29). The Endocrine Society clinical practice guideline also recommended that all patients with a hormonally functional PPGL should receive α-AR antagonists to prevent perioperative cardiovascular complications (1). It is recommended that even normotensive patients of PPGL should receive preoperative α-AR blockade to prevent potential risk of intraoperative hypertension. While for patients with parasympathetic-derived head and neck paragangliomas or biochemically silent PPGL, α-AR blockade may not be necessary, and there should be a collaborative decision with anaesthetist and surgeon, considering cardiovascular status and intraoperative risks of the patients (1, 10). However, there are some studies question the necessity of α-AR blockade, and report successful PPGL resection without this kind of drug. It is reported by some studies that there is no significant difference of perioperative blood pressure and hemodynamic stability with or without the use of α-AR antagonists (30–33). Especially in 2017, Groeben et al. (34) assessed hemodynamic conditions and perioperative complications in 110 patients with and 166 patients without α-AR blockade. Only a slight difference in mean maximal systolic pressure was reported between groups. There was no significant difference in the incidence of hypertensive episodes between patients with and without α-AR blockade, and no severe complications occurred in either group. It is so far the largest number of patients of PPGL without α-AR blockade reported, and this study demonstrated that surgery of PPGL without preoperative α-AR blockade is feasible. In 2019, a review about PPGL in New England Journal of Medicine (5) quoted this study and agreed that α-AR antagonists may be not necessary. Possible reasons for the idea of adrenergic receptor blockade-free management are as follows. Firstly, the guidelines (1, 29) which recommended necessity of α-AR antagonists for patients with PPGL stated that there was no RCTs or other high quality evidences available for this statement, and scientific investigations were unable to be carried out because of the very low incidence of the disease. Moreover, α-AR antagonists are not without adverse effects, and there are even significant side aspects sometimes (35). Secondly, in a patient with PPGL who has had side effects associated with long-term, episodic hypertension without α-AR blockade, it seems impossible that decompensation would occur several days before operation, and other short-acting anti-hypertensive drugs may be appropriate during aesthesia (5). Thirdly, with the improvement of diagnostic tools for identification and localization of the lesions, modern minimally invasive surgical techniques, and highly effective, short-acting drugs without severe side effects to control intraoperative conditions, the necessity of the unreliable, time-consuming preoperative management with potential significant adverse effects should be questioned (34).

## β-AR Antagonists

The use of β-AR antagonists is determined by the extent of CA-induced tachycardia or reflex tachycardia after the initiation of phenoxybenzamine. It is noteworthy that β-AR antagonists should never be treated alone or before adequate α-AR blockade. Since for patients with PPGL, loss of β-AR-mediated vasodilatation with unopposed CA-induced vasoconstriction can cause dramatic increase in blood pressure, or even hypertensive crisis (1, 12). Propranol, a non-selective β-AR antagonist, which is often used in the past, is given in doses of 10 mg, three to four times a day initially, and usually no higher than 200 mg/d. Nowadays, more cardio-selective β1-AR antagonists are recommended, such as atenolol (12.2–25 mg two to three times a day), metoprolol (25–50 mg two to three times a day), and metoprolol controlled release tables (Toprol) (25–200 mg once a day) (8). However, there is no ample evidence to support the use of β1-selective AR antagonists over non-selective AR antagonists in patients with PPGL (1). There are also some combined α and β-AR antagonists, such as labetalol and carvedilol. They have fixed ratio but more potent β than α antagonistic activities, such as α: β of labetalol is 1:5, but it is reported that α- to β-antagonistic activity should be at lease 4:1 to achieve sufficient antihypertensive effect. Therefore, they are not recommended as the initial therapy for PPGL to take the place of α and β-AR antagonists separately, to avoid episodes of hypertension or even hypertensive crisis (8). For patients with CA-induced cardiomyopathy, it is reported that β-AR antagonists can lead to severe hypotension, bradycardia and even cardiac arrest, which warrant extra caution (36).

## Calcium Channel Blockers (CCBs)

CCBs are the most commonly used drugs, in combination with α-AR antagonists, to further improve blood pressure control in patients with PPGL (1). Some studies consider this kind of drugs as the primary choice of preoperative management of PPGL, especially for normotensive patients or those with very mild hypertension, and for patients experiencing severe side effects with α-AR antagonists (8, 30, 37). These drugs can relax vascular smooth muscle and reduce peripheral vascular resistance by inhibiting NE-induced intracellular and transmembrane calcium influx in vascular smooth muscle (18). Compared to α-AR antagonists, CCBs do not cause drug-induced orthostatic hypotension and reflex tachycardia, and exert additional protective effects of the heart by preventing CA-mediated coronary vasospasm and myocarditis (38). A retrospective study showed no differences in intraoperative hemodynamic stability and short-term postoperative outcomes in patients with PPGL using nicardipine compared with phenoxybenzamine (39). Another study showed similar improvements in intraoperative hemodynamic instability between α-AR blockade and CCBs (40). It is reported in a study that the monotherapy of CCBs for the perioperative management of PPGL did not prevent all hemodynamic instability, but was associated with a lower morbidity and mortality (41). The most often used CCBs are nicardipine (60–120 mg per day), amlodipine (5–10 mg per day), and nifedipine (30–120 mg per day) (8). Nowadays, nifedipine controlled release tables (30–60 mg once a day) have become a good choice by its effective and long-acting profiles.

## CA Synthesis Inhibitor

It is proved that excessive CAs release results in perioperative cardiovascular instability in the patients with PPGL, so the administration of drugs which inhibit the CA biosynthesis may be beneficial for the treatment of PPGL (42). Metyrosine, a CA synthesis inhibitor, inhibits tyrosine hydroxylase, which catalyzes the conversion of tyrosine to dihydroxyphenylalanine (DOPA) (**Figure 1**), the rate-limiting step of the CA synthesis pathway (12, 43). Therefore, metyrosine has been used as one of the approaches for the management of PPGL, especially when phenoxybenzamine is unavailable in some areas, such as Italy. It takes effect at about 3 days and should be used at least 1–3 weeks before surgery, at initial dose of 500 mg per day and titrated as necessary to a maximum dose of 4 g per day (8, 44, 45). It was shown that a dose range of 1–2 g per day is well tolerated with relatively low incidence of side effects (46). Metyrosine can readily cross the blood-brain barrier, and inhibits CA synthesis in the brain as well as in the periphery, thus resulting in sedation, somnolence, anxiety, depression, and rarely leading to extrapyramidal signs, such as parkinsonism. It should also be used with caution for patients with renal dysfunction, since metyrosine is excreted through kidney and the pharmacokinetics of metyrosine are significantly affected by renal dysfunction. Most adverse effects would disappear after cessation of administration (8, 45). It was shown that urine MNs have been reduced for at least 50% from baseline after the administration of metyrosine (45, 46). Although Butz et al. reported that patients treated with metyrosine and pheoxybenzamine had wider range of intraoperative blood pressure variations than phenoxybenzamine-only patients (47), it has been reported by most studies that the combination of metyrosine and α-AR antagonists lead to better blood pressure control, decreased intraoperative blood loss, and reduced volume replacement during operation compared with the classical method of monotherapy of α-AR blockade (45, 46, 48, 49). In a case report of a patient with PPGL, the administration of metyrosine alone was unable to satisfactorily control intraoperative blood pressure (50), which was probably due to the incomplete depletion of CA stores no matter what dose used. Therefore, metyrosine is always used in combination with α-AR antagonists in patients with serious symptoms which cannot be well controlled by other medications, such as those with biochemically active tumors or extensive metastatic tumors (8, 45). However, the limited availability of this drug and its adverse effects at high doses limit its widely use (8).

## Cardiovascular Evaluation and Blood Volume Restoration

The excessive CAs release and resultant hypertension can lead to significant changes in the cardiovascular system, such as vasoconstriction of the coronary arteries, increased arterial stiffness, arrythmias, and cardiomyopathy (13). Moreover, it was shown that normotensive patients with PPGL had similar perioperative hemodynamic instability to those with significant preoperative hypertension (51). Therefore, it is essential to perform cardiovascular evaluation for every patient with PPGL. The evaluation should include a thorough history, physical examination, complete laboratory tests, electrocardiogram (ECG), and echocardiography. An ECG may reveal pathologic findings such as nonspecific ST-T wave changes, arrythmias, and signs of left ventricular hypertrophy, which may be related to the CA-induced coronary artery vasoconstriction that obstructs myocardial blood flow. An ecocardiography is recommended to evaluate the presence of cardiomyopathy, and may be helpful to determine improvement after therapy (13).

Restoration of blood volume decreases the risk of protracted hypotension or shock as a result of sudden vasodilation during surgery. However, the management with α-AR antagonists alone will lead to blood volume restoration in only approximately 60% patients with PPGL (8). Therefore, it is recommended by the Endocrine Society clinical practice guidelines that treatment of PPGL should also include fluid intake and a high-sodium diet to restore blood volume preoperatively and prevent perioperative hypotension, although evidence from RCTs is not available. A high-sodium diet (e.g., 5,000 mg per day) is usually initiated 3 days after the administration of α-AR blockade, and continuous saline infusion (e.g., 2,500 ml per day) is usually started in the evening before surgery. For patients with heart or renal failure, special caution is required for volume loading (1, 5).

## Other Recommendations

The administration of drugs that provoke the release of CAs produced by the tumor or interfere with CAs metabolism should be avoided for patients with PPGL. E and NE release can be provoked by steroids, histamine, glucagon, vasopressin, and angiotensin II. Drugs that are used for obesity management, such as phentermine, phendimetrazine, and phenylethylamine, are sympathomimetic amines with a direct action on adrenoceptors. NE reuptake inhibitors, such as tricyclic antidepressants and amitriptyline, cocaine, and those that interfere with NE metabolism, all result in high circulating NE levels, and should be avoided by PPGL patients (8). Moreover, strenuous physical activities, smoking, and alcohol consumption should also be avoided, since they all significantly increase CAs release from a tumor. To ensure optimal preoperative management for patients with PPGL, multidisciplinary teamwork, including endocrine, surgical, cardiology, anesthesia, and oncology teams, is essential (8).

## Is Preoperative Management Necessary for Patients With Clinically Silent and Biochemically Silent PPGL?

It was reported that patients with clinically silent (normotensive) but biologically active PPGL had relatively lower levels of CAs than those with hypertensive PPGL, since the expression of multiple genes which were involved in key processes of CA synthesis was decreased in these tumors (10, 52). Studies that evaluating the value of preoperative management for patients with normotensive PPGL are scarce. Although one study showed that preoperative α-AR antagonist had no benefit for patients with normotensive PPGL (31), another study revealed that normotensive PPGL had similar perioperative hemodynamic instability with hypertensive PPGL, which differed significantly from nonfunctioning adrenal adenomas (51). Therefore, as recommended by the Endocrine society, patients with normotensive PPGL should also receive α-AR antagonists or CCBs to prevent unpredictable hypertension during surgery (1).

For biochemically silent PPGL, which do not secret CAs, hemodynamic instability resulting from the tumors should not occur theoretically. However, malignant hypertension during surgery has been reported by several cases in patients with biochemically silent PPGL not receiving preoperative management (53–55). Mechanisms remain unclear. It is speculated that some could be a result of undetected dopamine secretion by the tumor, and others may originate from the intratumoral CA release during surgery (10). There is no consensus about the necessity of preoperative management for patients with biochemically silent PPGL, but it is recommended that before clinicians choose not to pre-medicate preoperatively, cardiovascular status and perceived intraoperative risks should be evaluated, and decision should be made by multidisciplinary teamwork (10).

## Management of Hypertensive Crisis

PPGL may cause potentially lethal hypertensive crisis due to the effects of the excessive released CAs. Hypertensive crisis is an acute, life-threatening situation associated with severe increase in blood pressure, requiring special attention. It is defined as a systolic blood pressure higher than 180 mmHg or a diastolic blood pressure higher than 120 mmHg, with or without acute target organ damage (56). Hypertensive crisis may develop as a consequence of postural changes, urination, emotional stress, and use of certain drugs which may provoke the release of CAs. It may also be induced by administration of a β-AR antagonist without sufficient α-AR blockade, or during a surgery without proper preparation. The clinical presentation may vary, including headaches, nausea, vomiting, visual disturbances, and palpitations. In this case, control of blood pressure may be achieved by a continuous infusion of sodium nitroprusside at 0.5–10.0 μg/kg/min, or phentolamine, a short-acting α-AR blockade, given as an intravenous bolus of 2.5–5 mg at the rate of 1 mg/min, which can be repeated every 3–5 min. Urapidil, a selective α1-AR antagonist, can also be used to control blood pressure during hypertensive crisis at the dose of 10–15 ml/h in continuous infusion, or as a bolus of 25 or 50 mg intravenously (57). If conventional antihypertensive treatments do not achieve optimal effects, magnesium sulfate can be used. It is an effective arteriolar dilator, and can inhibit the function of excessive CAs release. Magnesium sulfate should be administered with a loading dose of 40–60 mg/kg followed by an continuous infusion of 1–2 g/h (58).

## Conclusions

In summary, preoperative management of PPGL, which includes hypertension control and improvement of blood volume, is crucial. The most common approach is to block the function of excessive plasma CAs, and α-AR antagonists are the first choice. Hypertension may be slightly better controlled by non-selective α-AR antagonist, phenoxybenzamine, for some patients, at the cost of higher risk of postoperative hypotension and other side effects. While selective α-AR antagonist, doxazosin, is proved to have much less adverse effects, but is more likely to be used in combination with additional antihypertensive drugs. With the improvement of diagnostic tools for identification and localization of the lesions, modern minimally invasive surgical techniques, and highly effective, short-acting drugs without severe side effects, the necessity of the preoperative management of α-AR antagonists with potential significant adverse effects should be questioned. The use of β-AR antagonists is determined by the extent of CA-induced tachycardia or reflex tachycardia after the prescription of phenoxybenzamine. It is noteworthy that β-AR antagonists should never be treated alone or before adequate α-AR blockade. CCBs are the most often used drugs in combination with α-AR antagonists, to further improve blood pressure control in patients with PPGL. Some studies considered this kind of drugs as the primary choice of preoperative management of PPGL, especially for normotensive patients or those with very mild hypertension, and for patients with severe side effects when using α-AR antagonists. CA synthesis inhibitor, metyrosine, is used combined with α-AR antagonists to patients with serious symptoms which cannot be well controlled by other medications, such as those with biochemically active tumors or extensive metastatic tumors. It is essential to make cardiovascular evaluation for every patient with PPGL and it is recommended that treatment of PPGL should also include a high-sodium diet and fluid intake to restore blood volume preoperatively and prevent perioperative hypotension. However, PPGL is a rare disease, so large RCTs or meta-analyses are not available at present. Therefore, more convincing evidence is needed to determine the most proper preoperative management strategies.

## Author Contributions

FF, LD, QH, and ML conducted a review of the literature and wrote the review. FF and ML contributed to conception and design of the review, and ML finalized the review. All authors contributed to the article and approved the submitted version.

## Funding

This study was support by the National Key R&D Program of China (2019YFA0802502) and Natural Science Foundation of China (81830025 and 81620108004). We acknowledge support from Tianjin Municipal Science and Technology Commission (17ZXMFSY00150 and 18JCYBJC93900).

## Conflict of Interest

The authors declare that the research was conducted in the absence of any commercial or financial relationships that could be construed as a potential conflict of interest.
